# Association of FTO gene variants with body composition in UK twins

**DOI:** 10.1111/j.1469-1809.2012.00720.x

**Published:** 2012-07-23

**Authors:** Gregory Livshits, Ida Malkin, Alireza Moayyeri, Timothy D Spector, Christopher J Hammond

**Affiliations:** 1 Department of Twin Research and Genetic Epidemiology, King's College LondonUK; 2 Human Population Biology Research Unit, Department of Anatomy and Anthropology, Sackler Faculty of Medicine, Tel Aviv UniversityTel Aviv, Israel

**Keywords:** BMI, lean and fat body mass, waist and hip circumference, SNP, association analysis

## Abstract

The association of *FTO* gene variants with body mass index (BMI) and other obesity characteristics is well established. However, uncertainties remain whether the association is present only in young populations and whether it is attributable to body fat mass specifically. We aimed to clarify these two questions in a large sample (*N*= 4,523 individuals) of middle-aged and older (range 40–80 years) British female twins. The women were assessed for BMI, waist and hip circumference, total lean (LBM) and fat (FBM) body mass. Since the majority of *FTO* association signals have been reported in a haploblock bordering 52,355–52,408 kb (on chromosome 16q12.2), we examined five genotyped and 43 imputed SNPs mapped to this block. Canonical correlation and other association analyses showed significant and consistent association between the selected SNP and studied body composition phenotypes, with *p*-values reaching *p*= 0.000004. Of particular interest, in addition to the expected significant associations between *FTO* variants and FBM, we also identified significant associations with LBM. These results suggest that the association between *FTO* variants and body composition phenotypes is present across a wide range of ages, and that *FTO* appears primarily to affect the amount of body soft tissue, influencing both fat and lean mass.

## Introduction

**Figure 1:**
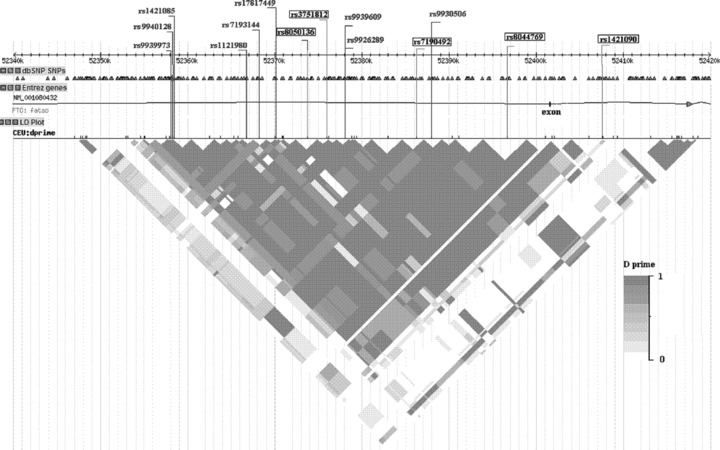
Location of significant results for *FTO* gene (16q12.2) variants reported previously as associated with BMI and obesity in relation to the five SNPs genotyped in this study (shown in rectangles). Data Source: HapMap Data Rel 27 PhaseII+III, Feb09, on NCBI B36 assembly, dbSNP b126. The majority of the previously reported significant results with BMI were mapped to the first haploblock: rs9939973, rs9940128, rs1121980, rs8050136 (Hinney et al., 2007; Scuteri et al., 2007), rs1421085 (Ho et al., 2010; Attaoua et al., 2009), rs1121980 (Hinney et al., 2007; Scuteri et al., 2007), rs7193144 (Hinney et al., 2007; Scuteri et al., 2007), rs17817449, rs3751812, rs8050136 (Ho et al., 2010), rs9939609 (Lopez-Bermejo et al., 2008; Rutters et al., 2011; Hinney et al., 2007), rs9926289, rs9930506 (Scuteri et al., 2007).

It is well established that interindividual variation of numerous and diverse body-composition and obesity phenotypes is governed to a substantial degree by genetic factors (Livshits et al., 1998; 2007; Farooqi & O’Rahilly, 2006; Farooqi, 2011). Regardless of the population studied, familial composition of the sample and method of data analysis, heritability estimates have commonly been reported as highly significant, and have often explained more than 50% of the phenotype variation. Nevertheless, it remains largely unknown which specific genes and functional DNA polymorphisms are involved. Several genetic-association studies conducted in recent years have consistently shown that the variants of the fat mass and obesity associated gene, *FTO*, are significantly associated with body mass index (BMI, kg/m^2^, see Fig. 1) and fat percentage variation (Frayling et al., 2007; Kilpeläinen et al., 2011) and with other obesity-related phenotypes (Fawcett & Barroso, 2010; Hertel et al., 2011). The association is most consistently found between the single nucleotide polymorphism (SNP) rs9939609 in the FTO gene, in both cross-sectional and longitudinal studies. These and other published data suggest, however, that there are several more SNPs significantly associated with BMI and other obesity phenotypes, and that they are mostly mapped to a single linkage disequilibrium (LD) block of about 50 kb, including the first two exons as well as the first intron (Fawcett & Barroso, 2010; Berulava & Horsthemke, 2010; Rutters et al., 2011).

There, are, however, unanswered questions. The first relates to the age-dependency of the *FTO* effect. This association is commonly seen in young adults and in children, and is not well replicated in elderly cohorts (Jacobsson et al., 2009; Jacobsson et al., 2011). The second relates to the phenotype specificity of effect. Although anthropometrical measurements such as BMI and waist to hip circumference ratio (WHR) are often used as indicators of obesity, they are in fact surrogate measures that also reflect other aspects of body composition, namely lean and bone mass, and not necessarily only fat mass. Indeed, implementing widely used DXA technology, one can quantitatively assess the major components of human body composition, including Fat Body Mass (FBM) and fat free Lean Body Mass (LBM), which consists mostly of muscle mass, but also includes soft tissue of internal organs and tendons.

The major aim of this study was to assess whether the aforementioned associations between *FTO* polymorphisms and BMI are reproducible in a large sample of middle-aged and older British women and, if so, whether it is attributable to fat (FBM) or nonfat lean (LBM) body mass.

## Materials and Methods

### Sample

The data examined in the present study are from the TwinsUK Adult Twin Registry, described in detail elsewhere (Spector & Williams, 2006). All participants gave written informed consent before entering the study and the St. Thomas’ Hospital research ethics committee approved the project. The volunteer sample was collected from the general population through national media campaigns in the UK and without first ascertaining the presence of any individual characteristics, diseases or traits. The present study is based on 4523 individuals, all female twins, collected since 1992. Each individual in the sample had one or more repeated DXA measurements of total lean (LBM) and fat (FBM) mass, and 3808 had genome wide association (GWAS) data. Measurements were carried out from 1992 to 2011. The sample used in the current analysis includes individuals aged 40 or over and consists of 1045 pairs of monozygotic (MZ) and 1121 pairs of dizygotic (DZ) twins and 191 single individuals, belonging to 18 MZ and 173 DZ sibships with only one sibling phenotype available. The age at which the included individuals were examined varied between 40 and 80 years. As the cohort is overwhelmingly of North European ancestry (98%), participants of other ethnicities were removed using standard principle component analyses.

### Phenotypes

The body composition components, including FBM and LBM, were measured using the standard whole body DXA method, following manufacturer's recommendations (QDR 4500W system, Hologic Inc, Bedford, MA). The subject lay supine on a bed and was scanned from head to toe for determination of total bone mineral density, total lean tissue mass (LBM), and total fat mass (FBM) as described in detail elsewhere (Arden & Spector, 1997). All scan printouts were reviewed by an expert reader to ensure proper positioning and analysis. Measurements of BMI (kg/m^2^), waist and hip circumferences (cm) were also made by trained research nurses.

### SNP Selection

The genotype data were based on genome-wide association scans performed in the TwinsUK cohort and were analysed using the Illumina (San Diego, USA) 317K and 610K SNP arrays, with a call rate of genotype ≥ 98%. Using the published data (Frayling et al., 2007; Rutters et al., 2011) and the International HapMap and UCSC browsers, the *FTO* region was identified where significant association results were largely restricted to a haploblock, positioned between 52,355 and 52,408 kb on chromosome 16q12.2 (Fig. 1). Five genotyped SNPs were available in this region and all were at Hardy–Weinberg equilibrium *p* > 0.01.

Four of the selected SNPs were in high and significant linkage disequilibrium, *D*′ > 0.89 (*R*^2^ > 0.62), with each other (Table 1, supplementary material). The fifth SNP, rs1421090, was in significant but low LD (*D*′∼ 0.10) with other markers. Marker rs9939609 was not available; however, according to HapMap data it is in strong LD with the first four SNPs included in this study (Fig. 1). Imputation of rs9939609, and an additional 42 SNPs in this haploblock with MAF > 0.05 and Hardy-Weinberg *p* > 0.01, was performed. The imputation quality and procedure is given elsewhere (Southam et al., 2011). Since by definition these data are derivatives of the genotyped SNPs, with which they are in high LD, to avoid redundancy in data presentation, the results of the corresponding analyses are given in the supplementary material.

**Table 1:** Basic descriptive statistics of the study phenotypes.

Trait	Descriptive statistic for traits and covariates						Correlation of traits after adjustment for age and height				
	
	MZ			DZ			FBM	LBM	Waist	Hip	WHR
*N*	Mean	SD	*N*	Mean	SD
BMI (kg/m**^2^**)	2108	25.7	4.6	2415	25.6	4.7	0.881	0.737	0.849	0.878	0.335
FBM (kg)	2108	24.2	8.2	2415	24.5	8.9		0.471	0.791	0.854	0.274
LBM (kg)	2108	39.1	5.4	2415	39.2	5.3			0.578	0.548	0.279
Waist (cm)^1^	1049	79.7	9.7	2006	80.5	10.6				0.799	0.673
Hip (cm)^1^	1049	101.5	8.5	2005	102.6	9.8					0.102
WHR	1049	0.784	0.057	2005	0.785	0.064					
Height (cm)^2^	2108	161.1	6.3	2415	162.2	6.1					
Weight (kg)	2108	66.8	12.1	2415	67.4	12.6					
Age (year)^2^	2108	54.3	8.2	2415	51.5	7.8					

1,2 The differences between the MZ and DZ twins were statistically significant, with *p* < 0.05 and *p* < 0.001, respectively.

In 18 MZ and 173 DZ sibships phenotype for only one sibling was available. However, all available (measured) individuals, including singletons, were included in this analysis.

### Preliminary Statistical Analysis

As well as descriptive statistics for each phenotype in the study, correlations and cross-correlations within and between the individuals and traits were estimated. In addition to regular Pearson correlations between the individual phenotypes, the trait correlations within the twin pairs were also computed for each zygosity. Differences between MZ and DZ correlations suggest the extent of potential involvement of genetic factors in phenotype variation. The cross-correlation between the traits and between the individuals (e.g., trait X in twin 1 and trait Y in twin 2) by twins’ zygosity gives an impression of the shared genetic factors effect for each pair of traits. Finally, to diminish the effect of multiple testing, canonical correlation analysis was conducted between the phenotypes and genotype scores (0, 1, 2) of each of the five SNPs simultaneously (Anderson, 1984). Three thousand and fifty individuals had complete phenotypic measurements and genotypes and were included in this analysis. The goal of this analysis is preliminary evaluation of the relationships between the two sets of latent variables: one set represents an array of dependent variables (body composition phenotypes) and the other is the set of independent predictors (genotypes). The canonical correlation is optimized to maximize the linear correlation between the two sets of variables. These tasks were carried out using the STATISTICA 7.1 package (http://www.statsoft.com).

### Genetic Association Analysis of FTO Gene Variants

The whole available sample (Table 1) was examined implementing regression-based association analysis between quantitative trait and each of the selected SNPs, using the FASTA (FAmily-based Score Test for Association) method (Chen & Abecasis, 2007) as implemented in GenABEL (Aulchenko et al., 2007). For twin pairs, the association was also tested implementing variance component analysis (VCA) of each of the study phenotypes, and genotype scores as covariates (Korostishevsky et al., 2010). In addition, VCA was used to obtain additive heritability estimates for each quantitative phenotype. All the dependent variables (except BMI) were adjusted for age (years) and body height (meters) prior to association analysis. BMI was adjusted for age only. Analyses used take into account the familial structure of the sample. The two methods of analysis are mutually complementary. In GenABEL, association analysis includes only actually genotyped individuals, e.g., one from each pair of MZ twins. In VCA, as implemented in the MAN_10 package (Malkin & Ginsburg, 2009), both members of a pair of MZ twins are included in the analysis where phenotypic data on both twins are available.

#### Appropriate significance Level

The purpose of this study was not a GWAS or a replication of the numerous previous publications showing association of *FTO* polymorphisms with BMI, rather it was to evaluate to what extent this association is attributable to LBM and/or FBM variation association with *FTO*. Given we have selected a small number of SNPs, *a priori* known to be associated with BMI and fat mass-related phenotypes, we obviously do not need genome-wide significance levels (all generally accepted significance values, i.e., *p* < 0.05, were considered significant).

## Results

Table 1 provides basic descriptive statistics of the participants with phenotypic data, with their corresponding sample sizes by twins’ zygosity. For the 4523 individuals included overall, mean age 52.8 (SD = 8.1 years), the mean BMI was 25.7 kg/m^2^ (SD = 4.7). As seen in the left-hand side of the table, MZ twins were slightly older then DZ twins (54.3 vs. 51.5 years), and tended to be shorter and to have smaller waist and hip circumferences. These phenotypic differences reached statistical significance (Table 1), but were attributable to age differences between the samples. There were no significant differences in relation to the rest of the body composition phenotypes; for example, mean BMI was 25.7 versus 25.6, and LBM 39.1 versus 39.2 for MZ and DZ twins. Correlations between different obesity-related traits were, as expected, statistically highly significant (*p* < 0.001) and of sizeable magnitude, presented in the right-hand section of Table 1 as pairwise correlation coefficients, after their adjustment for age and height (age only for BMI). BMI, FBM, waist and hip measures were most correlated, LBM and WHR less so. WHR was included as it has been previously used as an index of abdominal obesity. All within the trait twin correlations were highly significant (*p* < 0.001) and consistently almost exactly twice as high in MZ as in DZ twins, suggesting significant genetic influence, with heritability estimates for BMI of 0.73, FBM of 0.78 and LBM 0.57. Anthropometric phenotypes were similarly highly heritable (Table 2). Cross-correlations between the traits and twins were also statistically significant (at least *p* < 0.05) for all comparisons, other than WHR with hip circumference, and again generally twice as high in MZ as in DZ twins.

**Table 2:** Sibling correlations and cross-correlations for MZ and DZ twins (after adjustment on age and body height).

Traits	BMI	FBM	LBM	Waist	Hip	WHR
MZ	DZ	MZ	DZ	MZ	DZ	MZ	DZ	MZ	DZ	MZ	DZ
BMI	0.751	0.390										
FBM	0.659	0.344	0.757	0.393								
LBM	0.597	0.287	0.363	0.138	0.781	0.467						
Waist	0.623	0.326	0.554	0.297	0.398	0.24	0.692	0.354				
Hip	0.635	0.362	0.619	0.345	0.365	0.239	0.582	0.301	0.722	0.382		
WHR	0.294	0.095	0.202	0.074	0.231	0.098	0.465	0.209	0.122	0.031^(NS)^	0.629	0.298
*h***^2^**± SE	0.73 ± 0.05	0.78 ± 0.02	0.58 ± 0.06	0.71 ± 0.03	0.76 ± 0.03	0.66 ± 0.03						

NS: statistically nonsignificant correlation (*p* > 0.05); all the other correlations were statistically significant with *p*≤ 0.01.

There was, as expected, substantial pair-wise LD between the selected SNPs in the genomic region of interest (Fig. 1; Table S1, supplementary material). Canonical correlation analysis (Table S2, supplementary material) was therefore conducted to diminish the problems of data redundancy and multiple testing. The analysis showed that the two sets of variables (SNPs vs. body composition phenotypes) are not independent (by Bartlett's test *p*= 0.0005). Some 1.2% of the body composition variables (canonical root) were explained by the five SNPs. We attempted also to estimate the proportion of phenotypic variance, which can be explained by best (optimized) linear combination of SNPs (Table S2). This was done for each phenotype separately and for their best linear combination, which was most significantly correlated with linear combination of the SNPs. When the analysis was simplified for each quantitative trait versus five SNPs, the most significant canonical correlation was found with BMI (*R*= 0.104; *p*= 0.000004).

To clarify the specific pattern of the association, the association of all possible phenotype/SNP pairs was next tested separately (Table 3; see also Figure S1, supplementary material). The strongest associations were observed between the markers rs8050136 and rs3751812 and the phenotypes BMI, waist and hip circumference. These markers were also associated significantly with LBM and FBM, but the magnitude of association was lower. Waist and hip circumferences were significantly associated with all five SNPs, BMI and LBM with four SNPs, and FBM with three. WHR showed no significant associations. The implemented VCA allows one to test whether the effects of several SNPs, associated significantly with the specific phenotype, are independent of each other. However, in all instances using a likelihood ratio test, adding additional SNPs to rs8050136 or rs3751812 did not improve the data fitting, suggesting that the effects of the selected SNPs are not independent.

**Table 3:** Results of association analysis of the five selected SNPs in *FTO* gene with study phenotypes of body composition.

SNP_ID	Position (kb)	Allele	MAF	BMI (kg/m^2^)	LBM (kg)	FBM (kg)	Waist (cm)	Hip (cm)	WHR
rs8050136	52373776	A	0.393	0.47 ± 0.11^1^	0.35 ± 0.11	0.69 ± 0.21	1.14 ± 0.29	0.97 ± 0.27	0.004 ± 0.002
				[3.3E-05]^2^	[1.6E-03]	[8.9E-04]	[1.1E-04]	[3.6E-04]	[4.1E-02]
rs3751812	52375961	T	0.393	0.48 ± 0.11	0.36 ± 0.11	0.70 ± 0.21	1.16 ± 0.30	0.99 ± 0.27	0.004 ± 0.002
				[2.4E-05]	[1.3E-03]	[7.5E-04]	[8.8E-05]	[2.6E-04]	[4.3E-02]
rs7190492	52386253	A	0.377	−0.32 ± 0.12	−0.33 ± 0.11	−0.39 ± 0.21	−0.92 ± 0.30	−0.92 ± 0.28	−0.002 ± 0.002
				[5.0E-03]	[4.3E-03]	[6.9E-02]	[2.2E-03]	[8.3E-04]	[3.3E-01]
rs8044769	52396636	T	0.485	−0.42 ± 0.11	−0.34 ± 0.11	−0.58 ± 0.21	−1.25 ± 0.29	−0.94 ± 0.27	−0.005 ± 0.002
				[1.5E-04]	[1.9E-03]	[4.9E-03]	[1.9E-05]	[4.4E-04]	[6.0E-03]
rs1421090	52407671	G	0.268	−0.19 ± 0.12	−0.11 ± 0.12	−0.43 ± 0.23	−0.80 ± 0.32	−0.86 ± 0.29	−0.001 ± 0.002
				[1.3E-01]	[3.8E-01]	[6.0E-02]	[1.3E-02]	[3.5E-03]	[6.0E-01]

For each pair, SNP/phenotype,**^1^** regression coefficient beta ± SE (first line) and **^2^** *p*-value (second line, shown in parentheses) were calculated using the MAN package. The sample size for BMI, LBM and FBM was 4518 individuals; for waist and hip circumferences (and WHR) the number of valid subjects was 3055.

Next, we tested the combined effect of the alleles by comparing the +++++ homozygotes versus −−−−−homozygotes generated from the four SNPs in LD (rs3751812, rs7190492, rs8044769 and rs8050136). However, these tests did not change the results substantially (Table S3, see supplementary material). The corresponding *p*-values were of about the same magnitude as in Table 3. The association signals observed using the imputed SNPs were in full agreement with the above data, including rs9939609 (Table S4, supplementary material), although the strongest association (in terms of statistical significance) was observed with rs1121980.

Finally, we examined the effect of adjusting the BMI and circumference measurements for LBM and FBM on the strength of their corresponding *FTO* associations. The adjustment for LBM and FBM separately diminished the associations, but they remained significant (Table 4). Only simultaneous adjustment for both body composition components diminished the corresponding associations to an insignificant level (*p* > 0.05), suggesting that both FBM and LBM contribute to the associations observed with BMI, waist and hip circumferences.

**Table 4:** Results of association analysis of body mass phenotypes with the selected SNP. *P*-values for main phenotypes (BMI, waist and hip circumference), were adjusted preliminary for LBM or FBM, correspondingly.

SNPID	Allele	MAF	BMI_LBM	BMI_FBM	Waist_LBM	Waist_FBM	Hip_LBM	Hip_FBM
rs8050136	A	0.393	6.8E-03	2.5E-03	3.5E-02	1.5E-01	4.6E-02	3.5E-01
rs3751812	T	0.393	6.1E-03	2.2E-03	3.7E-02	1.4E-01	3.9E-02	2.8E-01
rs7190492	A	0.377	4.8E-01	7.8E-03	2.0E-01	4.7E-02	4.7E-02	2.2E-03
rs8044769	T	0.485	2.7E-02	2.7E-03	6.1E-03	4.4E-03	5.1E-02	1.1E-01
rs1421090	G	0.268	2.2E-01	7.4E-01	1.6E-01	7.9E-01	5.2E-02	4.5E-01

*P*-values were calculated using the GenAbel package.

## Discussion

Although, the amino acid sequence of the transcribed *FTO* protein is well established and clearly shows homology with the enzyme AlkB, which oxidatively demethylates DNA (Sanchez-Pulido and Andrade-Navarro, 2007; http://www.genecards.org/cgi-bin/carddisp.pl?gene=FTO), the exact physiological function of the *FTO* protein is not known. Therefore the specific molecular mechanism of its involvement in regulation of body composition and/or fat accumulation remains unclear. Nevertheless, the present study has replicated well the widely reported associations in young cohorts between *FTO* variants and anthropometrical indices of obesity, but in an older community-based sample of British women (aged 40 to 80, mean 52.8). We have also shown evidence that both lean and fat mass appear to underlie these associations. As expected from previous studies (Livshits et al., 2007; Herrera & Lindgren, 2010; Bogl et al., 2011), the phenotypes associated with obesity were highly heritable, with heritability estimates ranging from 0.58 ± 0.055 to 0.78 ± 0.02, after adjustment for covariates. These phenotypes, however, are not independent. Statistically significant cross-trait, cross-twin correlations (Table 2) suggested that common genetic and environmental factors potentially have a sizeable and significant effect on the covariation of virtually all the phenotypes. These findings suggest that the genetic polymorphisms identified for BMI might be expected to affect other body composition measurements. Indeed, significant associations were found between rs8050136 and rs3751812 and all the primary phenotypes studied, BMI, waist and hip circumference as well as LBM and FBM (Fig S1, supplementary material). Two other SNPs tested in this study, rs7190492 and rs8044769, demonstrated less consistent results, although rs8044769 was associated with waist circumference (*p*= 0.00005). Results for rs1421090, which was in low LD to other markers and located near the boundary of the study haploblock, were less significant, as might be expected. These results are in agreement with previously published data (Fig. 1; Haupt et al., 2008; Xi et al., 2010; Mangge et al., 2011) observed in young and adolescent populations of a very different ethnic background. Moreover, our results are also in accordance with Frayling et al.'s (2007) meta-analysis in which the authors reported a reliable association (*p*= 2E-4) between rs9939609 on BMI in elderly women (mean age 69 (SD = 5.5)) from the British Women's Heart and Health Study.

The findings obtained in the present study are comparable with published data, not only qualitatively but also in quantitative terms reflecting the size of the effect, despite the fact we studied a considerably older sample. For example, the estimated effect of rs8050136 on BMI per risk allele in this study was 0.48 ± 0.12 kg/m^2^, which is comparable to or even larger than for rs9939609 (0.28 kg/m^2^), as reported in a recent meta-analysis (Hertel et al., 2011). It seems that DNA associations in the first *FTO* haploblock are probably universal, independent of ethnic background and true for a wide range of ages.

The second question raised in this paper was: since *FTO* variants have been consistently associated with body mass related phenotypes, to what extent is this association attributable to FBM and/or LBM? Just before this paper was submitted for publication, Sonestedt et al. (2011) reported the results of their association analysis between rs9939609 and lean and fat mass in a very large sample (27,764) of unrelated individuals. Both associations as in this study were statistically highly significant (*p*= 2 × 10^−16^). Interestingly, the associations were attenuated but remained significant after adjusting for each other, suggesting existence of the potentially overlapping and nonoverlapping components in LBM and FBM variations, associated with *FTO* effect.

In our study, to answer this question, we adjusted the variation of BMI and waist and hip circumferences for LBM and FBM variations, separately and simultaneously, estimating extent of association with SNP of interest. When BMI was adjusted for LBM or FBM separately, the statistical significance of the association between the residual BMI variation and the selected SNP decreased but remained nominally significant (*p* < 0.05, Table 3). However, when BMI was adjusted for both body composition components, the association between the residuals and *FTO* variants became insignificant (*p* > 0.10). The SNP associations with LBM and FBM were of comparable magnitude; while the correlation between these two phenotypes was only moderate (*r*= 0.473). Our data therefore suggest that the *FTO* association with body size variation is mediated via both FBM and LBM, and not fat mass specifically. It should be mentioned that linear adjustment may not be the perfect way to test this hypothesis, as the adjustment to the expected population mean introduces some noise to residual variance. This in turn may affect the association, which after all explains only about 1% of the total variance of the phenotype.

The situation, however, is probably more complex and not unequivocal. A study reporting association between rs9939609 and several anthropometric traits of body mass (Mangge et al., 2011) found no association with circulating levels of conventional laboratory biomarkers of obesity, including adipokines such as leptin, adiponectin and resistin. Also, no independent associations were found between rs9939609 and rs8044769 and blood lipids or metabolic parameters related to insulin circulating levels and fasting glucose (Haupt et al., 2008; Xi et al., 2010). The current study found only marginally significant association between the selected SNPs and WHR, which is believed to reflect abdominal obesity (De Koning et al., 2007), while associations with waist and hip circumference (and BMI) variation were more significant. The lack of association with BMI adjusted for FBM and LBM, and a negligible association with body height (data not shown), as in other studies (Haupt et al., 2008; Xi et al., 2010), suggests *FTO* variants do not influence bone mass and growth. It is therefore possible that *FTO* mainly affects the amount of soft tissue, but appears to have less effect on metabolic markers relating to obesity, and WHR representing abdominal obesity.

In conclusion, the data obtained in this study confirm a significant association of *FTO* polymorphisms with BMI and other anthropometrical characteristics of obesity variation in a middle-aged and elderly community-based sample of British women. *FTO* is also significantly associated with LBM and FBM, and neither of these body composition measures separately explains *FTO* association with BMI. We therefore believe that *FTO* primarily affects the amount of body soft tissue, influencing both fat and lean mass. This inference has also found support in experiments with laboratory animals. For example, Gao et al. (2010) have shown significantly reduced LBM in *FTO* mutant mice and demonstrated that *FTO* plays an essential role in their postnatal growth. The authors’ assumption of key function of IGF1 in this process is consistent with the conclusion of this study that *FTO* has a role in regulating the total amount of body soft tissue, including both fat and lean.

## Supporting Information

Table S1 Linkage disequilibrium *D*′ between the study DNA markers **Table S2** Canonical correlation between the bodycomposition traits and DNA markers (*N* of minor allele) **Table S3** Body composition phenotypes' dependence on a number of positive effect alleles (relatively to BMI) in 4 SNPs (rs3751812, rs7190492, rs8044769, rs8050136) **Table S4** Main association results observed between thestudy body composition phenotypes and imputed SNPs in the firsthaploblock of the *FTO* gene (all available SNPs, including imputed). **Figure S1** Association (modulus of *p*-value decimallogarithm) of studied body composition phenotypes with the selectedSNP chromosomal position in *FTO* locus. See Table 3 for thespecific *p*-values and the size of the marker effect(regression coefficient).
