# Supplementary material for: Association of FTO gene variants with body composition in UK twins
Source: Ann Hum Genet. 2012 Jul 23;76(5):333–41. doi: 10.1111/j.1469-1809.2012.00720.x (PMC3532623; doi:10.1111/j.1469-1809.2012.00720.x)
Supplement: Table S1 — Linkage disequilibrium D′ between the study DNA markers Table S2 Canonical correlation between the bodycomposition traits and DNA markers (N of minor allele) Table S3 Body composition phenotypes' dependence on a number of positive effect alleles (relatively to BMI) in 4 SNPs (rs3751812, rs7190492, rs8044769, rs8050136) Table S4 Main association results observed between thestudy body composition phenotypes and imputed SNPs in the firsthaploblock of the FTO gene (all available SNPs, including imputed). Figure S1 Association (modulus of p-value decimallogarithm) of studied body composition phenotypes with the selectedSNP chromosomal position in FTO locus. See Table 3 for thespecific p-values and the size of the marker effect(regression coefficient). [file ahg0076-0333-SD1.doc]

**Supplementary material to manuscript:**

Association of FTO gene variants with body composition in UK twins, by Gregory Livshits, Ida Malkin,

Alireza Moayyeri, Timothy D Spector, Christopher J Hammond

*Table S1. Linkage disequilibrium D’ between the* study DNA markers

| | SNP_ ID | rs3751812 | rs7190492 | rs8044769 | rs1421090 | | --- | --- | --- | --- | --- | | rs8050136 | 0.9955 | 0.9879 | 0.9876 | 0.1073 | | rs3751812 |  | 0.9955 | 0.9906 | 0.1132 | | rs7190492 |  |  | 0.8885 | 0.0642 | | rs8044769 |  |  |  | 0.1352 | |
| --- | --- | --- | --- | --- | --- | --- | --- | --- | --- | --- | --- | --- | --- | --- | --- | --- | --- | --- | --- | --- | --- | --- | --- | --- | --- |

*Table S2. Canonical correlation between the body composition traits and DNA markers (N of minor allele)*

| N of Traits | Canon.  R | P-value | First set scores (phenotypes) | | | | | | Second set scores (DNA markers, N of minor allele) | | | | |
| --- | --- | --- | --- | --- | --- | --- | --- | --- | --- | --- | --- | --- | --- |
|  |  |  | BMI | FBM | LBM | Waist | Hip | WHR | rs8050136 | rs3751812 | rs7190492 | rs8044769 | rs1421090 |
| 6 ^1^ | 0.111 | 0.000559 | 0.321 | -0.005 | 0.432 | **-2.270** | 2.045 | 1.324 | -0.485 | **0.921** | -0.242 | -0.241 | -0.515 |
| 2 ^2^ | 0.106 | 0.000050 |  | -0.550 | **-0.654** |  |  |  | 0.381 | **-0.794** | 0.155 | 0.324 | 0.539 |
| 1 ^3^ | 0.104 | 0.000004 | 1.000 |  |  |  |  |  | 0.559 | **-1.127** | 0.006 | 0.317 | 0.489 |
| 1 | 0.087 | 0.000350 |  | 1.000 |  |  |  |  | 0.069 | -0.635 | 0.004 | 0.177 | **0.666** |
| 1 | 0.093 | 0.000080 |  |  | 1.000 |  |  |  | 0.613 | **-0.891** | 0.267 | 0.428 | 0.421 |
| 1 | 0.089 | 0.000190 |  |  |  | 1.000 |  |  | 0.181 | -0.416 | 0.009 | **0.674** | 0.436 |
| 1 | 0.099 | 0.000020 |  |  |  |  | 1.000 |  | 0.516 | **-0.866** | 0.336 | 0.169 | 0.602 |
| 1 | 0.055 | 0.104460 |  |  |  |  |  | 1.000 | -0.784 | 0.847 | -0.693 | **1.400** | -0.123 |

Canonical R**^2^,** similar to multivariate regression analysis, shows the proportion of the total variance of the studied array of phenotypes (body composition), attributable to the effect of the predictor variables (SNPs), and P-values reflect the significance of the canonical correlation.

^1^ 6 -The analysis is carried out for all six phenotypes simultaneously vs five selected SNP, ^2^ 2 - the same for FBM and LBM only, ^3^ 1 - the same for each variable separately. Number of valid cases in all analyses: 3050; BMI was adjusted for age; LBM, FBM, Waist, Hip and WHR were adjusted for age and height, prior to canonical correlation analysis. Canonical correlation analysis was used to examine the relations between the body composition phenotypes and the set of five selected SNPs. Canonical correlation is a multivariate procedure for assessing the linear relationship between two multidimensional (two sets) variables, called canonical variables, one representing a set of independent variables (five SNPs in this study), the other a set of dependent variables (body composition phenotypes). This procedure allows the simultaneous examination of the effect of several predictor variables (SNPs) on the criterion (outcome) variables. The logic here is that variables that are highly correlated with a canonical variate have more in common with it and they should be considered more important when deriving a meaningful interpretation of the related canonical variate. The criterion for choosing the important variables in each canonical variate is the structure coefficients (loadings). With canonical analysis, it is also possible to examine the correlation of each of the variables within the group of predictor variables (e.g. SNPs) to the outcome variables (all body composition phenotypes^1^ or for each of them^3^ separately).

Table S3. Body composition phenotypes dependence on a number of positive effect alleles (relatively to BMI) in 4 SNP (rs3751812, rs7190492, rs8044769, rs8050136)

| Phenotype* | Gr1 | | | Gr2 | | | Gr3 | | | t (Gr1 vs. Gr2) | | t(Gr1 vs Gr3) | | Correlation** | | |
| --- | --- | --- | --- | --- | --- | --- | --- | --- | --- | --- | --- | --- | --- | --- | --- | --- |
|  | N | Mean | Var. | N | Mean | Var. | N | Mean | Var. | t | p | t | p | R | N | P |
| FBM | 581 | 0.08 | 1.10 | 3227 | 0.00 | 1.00 | 458 | -0.04 | 1.09 | 1.66 | 4.9E-02 | 1.79 | 3.7E-02 | 0.045 | 3808 | 7.6E-03 |
| LBM | 581 | 0.08 | 1.03 | 3227 | -0.04 | 0.98 | 458 | -0.13 | 0.89 | 2.57 | 5.1E-03 | 3.49 | 3.0E-04 | 0.063 | 3808 | 8.0E-04 |
| BMI | 581 | 0.10 | 1.15 | 3227 | -0.02 | 0.98 | 458 | -0.10 | 0.95 | 2.41 | 7.9E-03 | 3.12 | 9.0E-04 | 0.066 | 3808 | 8.0E-04 |
| BMI_FBM | 581 | 0.07 | 1.03 | 3227 | -0.03 | 0.96 | 458 | -0.15 | 0.90 | 2.15 | 1.6E-02 | 3.45 | 3.0E-04 | 0.059 | 3808 | 9.0E-04 |
| BMI_LBM | 581 | 0.07 | 1.11 | 3227 | -0.01 | 0.99 | 458 | -0.01 | 0.92 | 1.59 | 5.6E-02 | 1.14 | 1.3E-01 | 0.035 | 3808 | 3.2E-02 |
| Hip | 407 | 0.13 | 1.20 | 2303 | -0.01 | 0.98 | 339 | -0.11 | 0.83 | 2.33 | 1.0E-02 | 3.28 | 5.0E-04 | 0.078 | 2710 | 8.0E-04 |
| Hip_FBM | 407 | 0.05 | 1.07 | 2303 | 0.00 | 1.00 | 339 | -0.11 | 0.94 | 0.84 | 2.0E-01 | 2.09 | 1.9E-02 | 0.045 | 2710 | 1.9E-02 |
| Hip_LBM | 407 | 0.06 | 1.12 | 2303 | 0.01 | 0.99 | 339 | -0.06 | 0.92 | 0.93 | 1.8E-01 | 1.60 | 5.5E-02 | 0.039 | 2710 | 4.5E-02 |
| Waist | 407 | 0.12 | 1.08 | 2304 | 0.00 | 1.01 | 339 | -0.11 | 0.88 | 2.18 | 1.5E-02 | 3.28 | 5.0E-04 | 0.077 | 2711 | 8.0E-04 |
| Waist_FBM | 407 | 0.04 | 1.00 | 2304 | 0.01 | 1.01 | 339 | -0.10 | 0.92 | 0.56 | 2.9E-01 | 2.02 | 2.2E-02 | 0.043 | 2711 | 2.7E-02 |
| Waist_LBM | 407 | 0.05 | 0.96 | 2304 | 0.02 | 1.02 | 339 | -0.06 | 0.94 | 0.56 | 2.9E-01 | 1.50 | 6.8E-02 | 0.035 | 2711 | 6.8E-02 |
| WHR | 407 | 0.05 | 1.001 | 2303 | 0.01 | 1.04 | 339 | -0.06 | 1.08 | 0.73 | 2.3E-01 | 1.37 | 8.5E-02 | 0.034 | 2710 | 7.5E-02 |

*All phenotypes were adjusted for age and height (except BMI), and then standardized prior to association analysis; _LBM & _FMM- means in addition adjusted for LBM or FBM correspondingly; Gr1 – individuals have 8 positive effect alleles in four- marker genotype; Gr2 – individuals have less than 8 positive alleles in four- marker genotype; Gr3 – individuals have 0 positive effect alleles in four- marker genotype. ** Correlation of the phenotype with the number of positive effect alleles in four- marker genotype.

*Table S4. Main* *association results observed between the study body composition phenotypes and imputed SNP in the*

*first haploblock of the FTO gene (all available SNP, including imputed).*

| SNP_ID | Position | MAF | HWE_p | P-values of the association test | | | | | |
| --- | --- | --- | --- | --- | --- | --- | --- | --- | --- |
|  |  |  |  | BMI | Waist | Hip | LBM | FBM | WHR |
| rs7206010 | 52312678 | 0.387 | 3.7E-02 | 6.9E-02 | 1.2E-01 | 9.0E-03 | 6.2E-02 | 3.0E-01 | 6.0E-01 |
| rs13333228 | 52351299 | 0.294 | 1.4E-02 | 1.9E-02 | 6.1E-02 | 3.0E-02 | 5.9E-03 | 2.5E-02 | 4.8E-01 |
| rs8047395 | 52356024 | 0.474 | 1.1E-02 | 6.2E-04 | 1.1E-04 | 2.9E-04 | 3.0E-03 | 1.5E-02 | 6.1E-02 |
| rs9937053 | 52357008 | 0.407 | 7.3E-01 | 1.9E-06 | 2.0E-04 | 2.0E-04 | 4.9E-05 | 5.4E-04 | 9.6E-02 |
| rs9928094 | 52357406 | 0.407 | 7.3E-01 | 1.9E-06 | 2.0E-04 | 2.0E-04 | 4.8E-05 | 5.4E-04 | 9.6E-02 |
| rs9930333 | 52357478 | 0.429 | 7.0E-01 | 3.5E-07 | 7.9E-06 | 6.0E-05 | 1.3E-05 | 1.4E-04 | 1.4E-02 |
| rs12446228 | 52357888 | 0.329 | 9.3E-02 | 9.2E-02 | 3.6E-02 | 5.5E-03 | 2.6E-02 | 5.1E-01 | 9.2E-01 |
| rs9939973 | 52358069 | 0.407 | 7.3E-01 | 1.9E-06 | 2.0E-04 | 2.0E-04 | 4.8E-05 | 5.4E-04 | 9.6E-02 |
| rs9940646 | 52358130 | 0.419 | 5.6E-01 | 5.2E-07 | 1.1E-04 | 7.6E-05 | 4.6E-05 | 2.0E-04 | 1.1E-01 |
| rs9940128 | 52358255 | 0.427 | 6.2E-01 | 1.2E-06 | 2.6E-05 | 4.4E-05 | 1.3E-05 | 4.9E-04 | 4.1E-02 |
| rs9923147 | 52359050 | 0.427 | 6.2E-01 | 1.5E-06 | 3.1E-05 | 5.4E-05 | 1.5E-05 | 5.8E-04 | 4.2E-02 |
| rs9923544 | 52359486 | 0.427 | 6.2E-01 | 1.6E-06 | 3.2E-05 | 5.6E-05 | 1.5E-05 | 6.0E-04 | 4.2E-02 |
| rs8055197 | 52360657 | 0.481 | 2.8E-01 | 4.4E-03 | 1.6E-04 | 2.0E-03 | 1.1E-02 | 7.3E-02 | 1.2E-02 |
| rs1558902 | 52361075 | 0.394 | 5.3E-01 | 1.2E-05 | 1.9E-04 | 2.1E-04 | 1.9E-04 | 1.7E-03 | 9.9E-02 |
| rs1861866 | 52361841 | 0.482 | 4.0E-01 | 7.3E-03 | 2.6E-04 | 2.7E-03 | 1.4E-02 | 1.0E-01 | 1.6E-02 |
| rs10852521 | 52362466 | 0.494 | 7.6E-01 | 3.6E-04 | 3.1E-04 | 1.8E-03 | 3.1E-03 | 7.1E-03 | 3.2E-02 |
| rs11075985 | 52362708 | 0.429 | 6.8E-01 | 2.0E-06 | 1.6E-05 | 7.6E-05 | 1.6E-05 | 5.9E-04 | 2.0E-02 |
| rs9922047 | 52363781 | 0.483 | 5.7E-01 | 3.2E-03 | 9.8E-05 | 2.7E-03 | 7.6E-03 | 4.7E-02 | 5.2E-03 |
| rs17817288 | 52365265 | 0.486 | 7.5E-01 | 1.2E-02 | 8.4E-04 | 5.0E-03 | 1.2E-02 | 1.6E-01 | 3.5E-02 |
| rs1477196 | 52365759 | 0.312 | 2.6E-01 | 1.6E-02 | 6.8E-03 | 1.7E-03 | 1.8E-03 | 1.5E-01 | 5.2E-01 |
| **rs1121980** | 52366748 | 0.426 | 8.1E-01 | 7.3E-08 | 2.2E-06 | 1.4E-05 | 6.4E-06 | 3.4E-05 | 1.2E-02 |
| rs7193144 | 52368187 | 0.393 | 4.3E-01 | 9.2E-06 | 1.1E-04 | 2.5E-04 | 2.7E-04 | 1.2E-03 | 5.5E-02 |
| rs8057044 | 52370115 | 0.479 | 7.3E-01 | 2.6E-05 | 1.2E-06 | 5.9E-05 | 3.7E-04 | 1.4E-03 | 1.9E-03 |
| rs17817449 | 52370868 | 0.394 | 5.2E-01 | 1.2E-05 | 1.3E-04 | 2.9E-04 | 4.3E-04 | 1.1E-03 | 5.9E-02 |
| rs8043757 | 52370951 | 0.395 | 4.7E-01 | 1.4E-05 | 1.2E-04 | 3.2E-04 | 5.3E-04 | 1.1E-03 | 5.1E-02 |
| rs11075987 | 52372662 | 0.494 | 7.3E-01 | 5.8E-04 | 7.9E-06 | 1.6E-04 | 4.7E-03 | 1.4E-02 | 4.6E-03 |
| rs8050136 | 52373776 | 0.393 | 6.1E-01 | 3.6E-05 | 1.9E-04 | 3.9E-04 | 1.1E-03 | 1.8E-03 | 6.2E-02 |
| rs4783819 | 52374148 | 0.365 | 3.7E-01 | 7.1E-02 | 7.2E-03 | 1.5E-03 | 2.0E-02 | 3.8E-01 | 5.1E-01 |
| rs8051591 | 52374253 | 0.393 | 6.1E-01 | 2.5E-05 | 1.5E-04 | 3.2E-04 | 7.2E-04 | 1.6E-03 | 5.9E-02 |

Table S4 (continuation).

| SNP_ID | Position | MAF | HWE_p | P-values of the association test | | | | | |
| --- | --- | --- | --- | --- | --- | --- | --- | --- | --- |
|  |  |  |  | BMI | Waist | Hip | LBM | FBM | WHR |
| rs9935401 | 52374339 | 0.393 | 6.1E-01 | 2.5E-05 | 1.5E-04 | 3.2E-04 | 7.2E-04 | 1.6E-03 | 5.9E-02 |
| rs3751812 | 52375961 | 0.393 | 7.3E-01 | 2.7E-05 | 1.7E-04 | 2.7E-04 | 8.4E-04 | 1.6E-03 | 7.2E-02 |
| rs3751813 | 52376209 | 0.429 | 9.4E-01 | 2.3E-03 | 2.6E-03 | 4.2E-04 | 1.2E-04 | 5.3E-02 | 5.4E-01 |
| rs9936385 | 52376670 | 0.382 | 2.0E-01 | 2.4E-05 | 2.2E-05 | 1.7E-04 | 3.9E-05 | 9.5E-04 | 1.6E-02 |
| rs11075989 | 52377378 | 0.407 | 8.4E-01 | 5.1E-06 | 2.8E-05 | 1.9E-04 | 1.5E-04 | 3.9E-04 | 2.1E-02 |
| rs11075990 | 52377394 | 0.408 | 8.6E-01 | 4.7E-06 | 2.9E-05 | 1.8E-04 | 1.2E-04 | 4.0E-04 | 2.3E-02 |
| **rs9939609** | 52378028 | 0.407 | 7.8E-01 | 3.3E-06 | 3.8E-05 | 1.9E-04 | 9.5E-05 | 3.0E-04 | 2.7E-02 |
| rs7202116 | 52379116 | 0.408 | 7.5E-01 | 4.9E-06 | 1.2E-04 | 3.4E-04 | 8.8E-05 | 5.1E-04 | 4.7E-02 |
| rs7201850 | 52379363 | 0.437 | 9.7E-01 | 1.9E-07 | 1.7E-06 | 7.2E-05 | 5.6E-06 | 3.8E-05 | 3.0E-03 |
| rs7185735 | 52380152 | 0.408 | 7.5E-01 | 2.1E-06 | 4.6E-05 | 1.8E-04 | 8.1E-05 | 1.9E-04 | 3.4E-02 |
| rs9941349 | 52382989 | 0.420 | 1.0E+00 | 7.0E-07 | 4.3E-06 | 3.3E-05 | 2.4E-05 | 1.5E-04 | 1.3E-02 |
| rs9931494 | 52384680 | 0.421 | 9.5E-01 | 8.9E-07 | 4.8E-06 | 6.4E-05 | 3.7E-05 | 1.5E-04 | 7.7E-03 |
| rs17817964 | 52385567 | 0.403 | 4.9E-01 | 6.9E-06 | 4.0E-05 | 1.1E-04 | 1.4E-03 | 2.4E-04 | 3.4E-02 |
| rs7190492 | 52386253 | 0.377 | 4.3E-01 | 1.5E-02 | 5.7E-03 | 1.1E-03 | 4.3E-03 | 1.6E-01 | 5.2E-01 |
| rs9930501 | 52387953 | 0.431 | 9.2E-01 | 1.5E-06 | 8.5E-06 | 5.2E-05 | 4.0E-05 | 3.6E-04 | 1.4E-02 |
| rs9930506 | 52387966 | 0.431 | 9.2E-01 | 1.5E-06 | 8.5E-06 | 5.2E-05 | 4.0E-05 | 3.6E-04 | 1.4E-02 |
| rs9922708 | 52388647 | 0.431 | 9.2E-01 | 1.7E-06 | 8.1E-06 | 5.6E-05 | 3.9E-05 | 4.3E-04 | 1.3E-02 |
| rs8044769 | 52396636 | 0.485 | 3.5E-01 | 3.8E-04 | 2.7E-05 | 4.7E-04 | 2.2E-03 | 1.2E-02 | 8.4E-03 |
| rs12149832 | 52400409 | 0.406 | 8.3E-01 | 1.7E-04 | 1.7E-04 | 4.4E-04 | 1.4E-03 | 8.4E-03 | 5.0E-02 |
| rs11642841 | 52402988 | 0.320 | 1.2E-02 | 9.7E-04 | 2.5E-04 | 4.2E-05 | 2.3E-02 | 5.5E-03 | 2.2E-01 |
| rs1421090 | 52407671 | 0.268 | 4.3E-02 | 1.2E-01 | 9.9E-03 | 3.2E-03 | 3.3E-01 | 5.5E-02 | 5.1E-01 |
| rs16953047 | 52687671 | 0.155 | 2.9E-01 | 6.3E-01 | 1.0E-01 | 9.7E-01 | 1.1E-01 | 5.0E-01 | 2.7E-03 |
| rs1071501 | 52689182 | 0.159 | 3.6E-01 | 6.9E-01 | 1.0E-01 | 9.5E-01 | 1.6E-01 | 4.9E-01 | 1.9E-03 |
| rs718388 | 52690401 | 0.158 | 6.2E-01 | 5.8E-01 | 1.4E-01 | 9.7E-01 | 1.7E-01 | 2.8E-01 | 5.9E-03 |
| rs12927155 | 52691301 | 0.154 | 4.5E-01 | 4.8E-01 | 1.2E-01 | 8.8E-01 | 9.6E-02 | 2.9E-01 | 5.9E-03 |
| rs12445828 | 52692303 | 0.158 | 6.2E-01 | 5.0E-01 | 1.1E-01 | 9.5E-01 | 1.5E-01 | 2.2E-01 | 3.7E-03 |
| rs12931414 | 52692494 | 0.158 | 6.2E-01 | 5.0E-01 | 1.1E-01 | 9.4E-01 | 1.5E-01 | 2.1E-01 | 3.8E-03 |
| rs2540775 | 52694760 | 0.154 | 4.5E-01 | 4.9E-01 | 1.2E-01 | 9.5E-01 | 9.7E-02 | 2.8E-01 | 5.0E-03 |
| rs2689264 | 52695481 | 0.156 | 3.2E-01 | 5.5E-01 | 1.1E-01 | 8.1E-01 | 1.3E-01 | 3.2E-01 | 6.5E-03 |

P-values were calculated using the GenAbel package.

*Figure S1. Association (modulus of P value decimal logarithm) of studied body composition phenotypes*

*with the slected SNP chromosomal position in FTO locus. See Table 3 for the specific p-values*

*and the size of the marker effect (regression coefficient).*
